# FODMAP Intake in Spanish Population: Open Approach for Risk Assessment

**DOI:** 10.3390/ijerph17165882

**Published:** 2020-08-13

**Authors:** Jonatan Miranda, Maialen Vázquez-Polo, Gesala Pérez-Junkera, María del Pilar Fernández-Gil, María Ángeles Bustamante, Virginia Navarro, Edurne Simón, Olaia Martínez

**Affiliations:** 1 Gluten Analysis Laboratory of the University of the Basque Country, Department of Nutrition and Food Science, University of the Basque Country, 01006 Vitoria, Spain; jonatan.miranda@ehu.eus (J.M.); maialen.vazquez@gmail.com (M.V.-P.); gesala.p@gmail.com (G.P.-J.); mariadelpilar.fernandez@ehu.eus (M.d.P.F.-G.); marian.bustamante@ehu.eus (M.Á.B.); virginia.navarros@ehu.eus (V.N.); olaia.martinez@ehu.eus (O.M.); 2 GLUTEN3S Research Group, Department of Nutrition and Food Science, University of the Basque Country, 01006 Vitoria, Spain

**Keywords:** dietary risk, national FODMAP intake, FODMAP risky food, low-FODMAP diet, FODMAP intake by age, underage FODMAP intake, open approach

## Abstract

Fermentable oligo-, di- and monosaccharides and polyols’ (FODMAP) were related with intestinal complications. The present study aimed to determine the FODMAP consumption of Spanish children, adolescents and adults, analyzing the real FODMAP risk of foods, and to set an open methodology for the measurement of this intake in other regions as well as nutrient intake assurance. Total fructan analysis was performed analytically in eighty-seven food samples. Daily intake of FODMAPs, fiber and micronutrients was calculated by combining the food composition for selected fermentable carbohydrates with the national food consumption stratified by age in an open software. Spanish child and adolescent total FODMAP consumption was settled as 33.4 ± 92.4 and 27.3 ± 69.0 g/day, respectively. Both intakes were higher than that of the adult population (21.4 ± 56.7 g/day). The most important food sources of lactose, excess of fructose and total fructan, considering their content and dietary intake were different between age groups. The contribution of these foods to dietary calcium and fiber and the consequent risk of deficiency if they are withdrawn was highlighted. We demonstrated the relevance of stratifying the total FODMAP intake by age. An open approach for FODMAP intake quantification and nutrient control was provided.

## 1. Introduction

Highly Fermentable oligo-, di- and monosaccharides and polyols (FODMAPS), are common dietary compounds. These 1–10 sugar-containing molecules are osmotically active substances that are poorly absorbed and which remain in the lumen of the intestine, acting as substrate for bacterial fermentation and producing short-chain fatty-acids and abdominal distention due to gas release [1].

FODMAPs have been blamed for their role as triggering agents of intestinal symptoms such as abdominal pain and bloating [2,3,4,5]. Their restriction in pathologies such as Intestinal Bowel Syndrome (IBS), Celiac Disease (CD) or Non-Celiac Wheat/Gluten Sensitivity (NCWS) have reported irregular results [6,7,8] and, though encouraging, this remains a subject of further investigation. It seems essential to determine the FODMAP intake of the population in order to evaluate its risk in terms of exposure, and to put in place effective strategies for diet-restriction when necessary, avoiding nutrient deficiencies, and depending on dietary habits and age [9,10]. This fact is also relevant in the case of pediatric population, as up to 20% is affected by functional bowel disorders (FBD) in the United States and Europe [11]. Nevertheless, little information about FODMAP consumption has been reported to date [12].

In this context, it is of interest to determine the intake of foods with symptom-triggering potential in the underage collective. The aim of the present study was to determine the FODMAP consumption of the Spanish adolescent and pediatric collective, as well as in adult population, and to set a methodology for the measurement of this intake, along with nutrient deficiency control, in other regions.

## 2. Materials and Methods 

### 2.1. Study Sample and Design

The present cross-sectional, descriptive and quantitative research took data from two National Dietary Surveys carried out in Spain: “Encuesta Nacional de Alimentación en la población Infantil y Adolescente” (ENALIA) and “Encuesta Nacional de Alimentación en la población adulta, mayores y embarazadas” (ENALIA 2). Both cross-sectional surveys are included in the European Food Safety Agency (EFSA) Comprehensive European Food Consumption Database [13], followed the deontological standards recognized by the Declaration of Helsinki and were carried out in accordance with the EFSA “EU Menu” guidance recommendations [14]. ENALIA study is a survey conducted in Spain from November 2012 to July 2014 designed to measure food consumption of children and adolescents [15]. By contrast, ENALIA2 collected information about eating habits and physical activity in adults (18–64 years old), elderly (65 to 74 years old) and pregnant women from June of 2014 to July of 2015 [16]. 

A representative sample of Spanish children (3–9 years old) (*n* = 825), adolescents (10–17 years old) (*n* = 745) and adults (18–74 years old) (*n* = 933) participated in the surveys. The study population was people living in households throughout Spain. The criterion of exclusion was the institutionalized population not resident in households, in the age ranges stated before. 

### 2.2. Dietary Assessment

In the mentioned ENALIA studies, dietary information was collected using two non-consecutive one-day food diaries (3–10 years old) or two 24 h dietary recalls (11 years and older) separated by at least 14 days. The 24 h recall was complemented by a Food Propensity Questionnaire including 1020 codified food items, designed specifically for this purpose [15].

### 2.3. Total Fructan Analysis

Fructo-oligosaccharides (FOS) and fructan polysaccharide-total fructan, analysis was performed by means of the commercial kit, K-FRUCHK (Megazyme International, Wicklow, Ireland) based on the Association of Official Agricultural Chemists (AOAC) method 999.03 and American Association for Cereal Chemists (AACC) Method 32.32. Eighty-seven samples containing cereal products plus onion and garlic were purchased from local supermarkets. Cereal-based product selection followed the consumption data of Spanish National Surveys (ENALIA and ENALIA2). Only frequently consumed, well-known brands were included in the sampling. Once in the laboratory, samples were stored until analysis at room temperature or frozen at −18 °C according to the storage conditions of the manufacturer.

### 2.4. Determination of FODMAP Daily Intake

Following the international recommendation by Varney et al. for cut-off values of high FODMAP diet [17], daily intake quantification was performed for total fructan, galacto-oligosaccharides (stachyose and raffinose), polyols (sorbitol and mannitol), excess of fructose (calculated subtracting glucose content to fructose content), and lactose. With the exception of fructans, the rest of the FODMAP concentrations (g per 100 g of food) were obtained from the open-access Food Composition Database named Food Standards Australia New Zealand [18].

The daily intake of each FODMAP was calculated by combining the food composition for selected fermentable carbohydrates with the national consumption stratified by age (3–9 years; 10–17 years; 18–74 years). Moreover, a top ten of those foods was created, representing the higher intake sources of lactose, excess fructose and total fructan in Spanish diet of each cohort. For this procedure, an open free software designed by the GLUTEN3S research team was used (GlutenFreeDiet) [19]. The software was linked to the Spanish food-composition database (BEDCA) integrated in the European Food Information Resource, and to the database with the FODMAP content, as previously detailed.

As the Australian database mentioned does not provide information about total fructan (only inulin), research articles were used as a reference for total fructan content of vegetables and fruits [20], as well as for pulses [21]. In the cases of cereal-based foodstuffs, onion and garlic, data from own determinations were used. Data on FODMAP content were uploaded to the previously mentioned software.

### 2.5. Determination of the Contribution of Main Risky Foods to Fiber and Micronutrient Daily Intake

The database generated in the open access software with national consumption food surveys for Spanish children, adolescents and adults could assess the daily intake of fiber, vitamin A, vitamin C, vitamin D, riboflavin, thiamin, calcium, magnesium, zinc, phosphorus and iron [15,16,19]. In order to evaluate the relevance of the top ten foods representing the higher intake source of lactose, excess fructose and total fructan to previous nutrients’ intake, these were subtracted from the previous database. Fiber and micronutrients content data were expressed as percentage of diet contribution.

### 2.6. Statistics

Variables, all quantitative, were presented as means ± standard deviations. Assuming the food-intake variables’ normality, due to the equality geometric and arithmetic mean of them, Student’s *t*-test (2-tailed unpaired *t*-tests) for independent samples was executed in order to compare FODMAP daily intake between Spanish children vs. adolescents, Spanish children vs. adults and Spanish adolescents vs. adults, as well as two different countries. Fisher’s exact test was used for comparison of each FODMAP proportion among different age-ranges. The level of statistical significance was fixed as *p* < 0.05. Data analysis was carried out using the IBM SPSS statistical package, version 24 (IBM Inc., Armonk, NY, USA).

## 3. Results

### 3.1. FODMAP Consumption of Spanish Children, Adolescents and Adult Population

Table 1 shows the FODMAP consumption of Spanish population, stratified by age. The total FODMAP intake ranged from 21.4 to 33.4 g/d, and was especially higher in the case of children (statistically significant only comparing to adults, *p* value < 0.01), because of their remarkable lactose consumption (*p* value of 0.06 and <0.001, comparing with adolescents and adults, respectively). In the case of fructan, teenagers reported a tendency towards higher intake than other age ranges (*p* value of 0.15 and 0.08, comparing with children and adults, respectively). By contrast, other FODMAPs such as excess fructose, stachyose, raffinose and mannitol increased with age; however, they did not always reach statistical significance.

**Figure 1:**
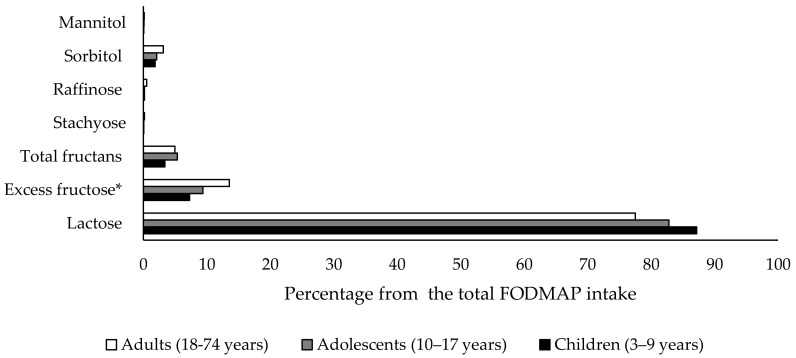
Influence of lactose, excess fructose, total fructan, stachyose, raffinose, sorbitol and mannitol to the total FODMAP intake of Spanish population in different age-range.

The influence of each compound to the total FODMAP intake was also calculated (Figure 1). Lactose input showed a tendency toward reduction over lifetimes (87%, 82% and 77% in children, adolescents and adults, respectively) while that of excess fructose (7%, 9% and 13%, respectively) or total fructan (3%, 5% and 5%, respectively) tended to become more important with age. By contrast, sorbitol, raffinose, staquiose and mannitol contributions to total FODMAP consumption stayed constant among groups (2% in the case of sorbitol and less than 1% in all the age-ranges for raffinose, staquiose and mannitol). The comparison of total FODMAP distribution among the analyzed age-range of Spanish population did not show statistical significance.

### 3.2. FODMAP-Risky Foods among Spanish Children, Adolescents and Adult Population

Table 2, Table 3 and Table 4 show the top ten foods representing the higher intake source of most abundant FODMAPs—lactose, excess fructose and total fructan—among the three age groups. Milk and yogurts were the main lactose sources in the diet, reflecting whole milk consumption habits in children and adolescents (Table 2 and Table 3), whereas skimmed or partly skimmed milk was the most important lactose intake source in adults (Table 4). An excess of fructose consumption was due to fruits such as apple or pear, white bread, tomato and honey among children and adolescents (Table 2 and Table 3). Apart from those foods, in the case of adult population, alcoholic beverages such as sweet white wine contributed notably to the consumption of this carbohydrate (Table 4). With regard to total fructan intake, cereals and vegetables made up the entire top ten classification of most consumed food in children and adolescents (Table 2 and Table 3). Furthermore, fruits such as peach, watermelon or melon were also relevant contributions in Spanish adult population (Table 4). White bread and plain sweet biscuits not only made a major contribution to excessive fructose intake, but also to total fructan intake in all the age ranges analyzed (Table 2, Table 3 and Table 4). Similarly, sponge cake was positioned as one of the most consumed foodstuffs for lactose and total fructan source, but only in Spanish children or adults in the case of lactose (Table 2, Table 3 and Table 4). 

Sorbitol and mannitol consumption was due to the same fruits, vegetables and fungi in the whole population (data not shown). Soy beverage as well as haricot and broad beans were the sole contributors of staquiose and raffinose intake in Spanish children, adolescents and adults (data not shown).

### 3.3. Fiber and Micronutrients’ Potential Deficiency Risk in a Low-FODMAP Diet Approximation

Risky food withdrawal from evaluated diet reflected the important contribution of these foods to dietary micronutrients and fiber. As expected, all the vitamins and minerals evaluated showed a decrease in children, adolescents and adults, but this was most remarkable for calcium, with a reduction of more than half (Table 5). For vitamins, vitamin C and riboflavin showed the highest depletion. Fiber was diminished noticeably in all the age ranges, but especially in children and adults, where it reached 55%.

### 3.4. Total FODMAP Consumption in Other Countries

**Figure 2:**
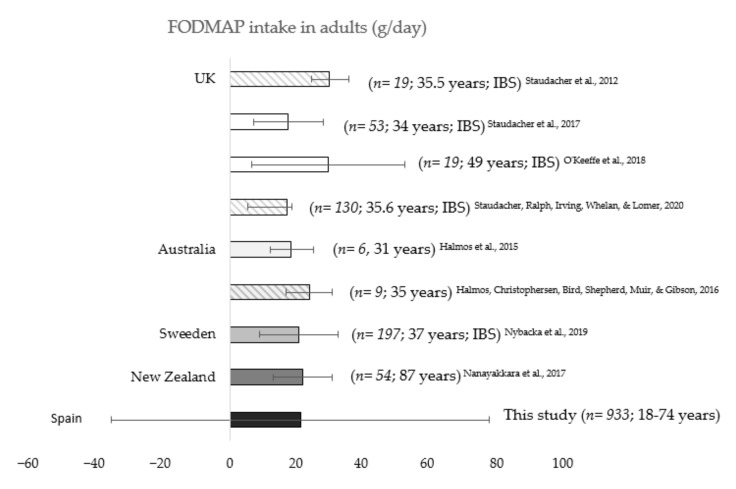
FODMAP intake of adult population in different countries (*n*; age; pathology). Columns corresponding to each country are depicted in different background color. Error bars in striped columns show a 95% confidence interval and those in plain columns show the standard deviation.

A review of total FODMAP consumption from adult population of other countries was performed and data were compared to those obtained in our study (Figure 2). Only two studies by Halmos et al. in Australia were carried out with healthy subjects [7,22]. Data revealed that the FODMAP consumption of participants was similar to that observed in our study for Spanish adult population (18.2 and 23.7 g/day). A direct comparison carried out between Spanish adults’ total FODMAP intake and a Swedish adult cohort, showed no differences between the two countries (20.7 g/day in Sweden vs. 21.1 g/day in Spain, *p* = 0.84) [23].

Other studies carried out in people with IBS, however, reported differing data, even in the same country. For instance, whereas Staudacher et al. and O’Keeffe et al. observed that the adult population consumed almost 30 g/day of FODMAPs in United Kingdom in 2012 [24] and 2018 [25] (29.6 and 29.4 g/day in 2017 and 2019, respectively), the same authors in 2017 [26] and 2020 [27], reported lower FODMAP ingestion in other studies conducted with United Kingdom adult citizens (17.4 and 17.0 g/day in 2017 and 2020, respectively).

## 4. Discussion

FODMAPs are a heterogeneous group composed of lactose, fructose, sorbitol, mannitol, galacto-oligosaccharides (GOS), and FOS and fructan polysaccharides (the last two making up total fructans), among others [17]. Classification is not so simple in reality, as fructose is only considered a FODMAP when it is in excess in relation to glucose, because then its active transportation is saturated, and it is hardly absorbed and may potentially ferment [28]. These properties must be complied with to be part of this group of molecules. Therefore, fructose should not be considered when it is free in the whole diet but rather when it is free in a meal or for a short period of time, making its intake difficult to measure.

In this respect, considerable efforts have been made in recent years to determine the FODMAP content of foods and to establish cut-off values for their classification in low- and high-content food [17]. Nevertheless, there is still a lack of information about composition of food products in different regions and so it is difficult to establish an accurate FODMAP intake worldwide [29]. Moreover, some FODMAPs, such as fructans, are more difficult to find in food composition databases, and their intake is not always determined in diet-related studies [6,22,24,25,30].

Interest in describing the intake profile of these dietary components lies in their potential role as factors for intestinal symptoms. Consequently, their dietary control is being investigated as a possible therapy in intestinal disorders (IBS, CD or NCWS) [31,32]. Children with FBD could also potentially benefit from a low-FODMAP diet (LFD). Based on epidemiological data [11], up to nearly one and a half million Spanish children and adolescents could benefit from limiting this intake, as they suffer some kind of intestinal symptom (functional dyspepsia, functional abdominal pain, abdominal migraine, or IBS) [33].

In view of the above, this work aims to fill the gap and calculate the total FODMAP intake for Spanish underage population, with the aid of a specific software containing information about FODMAP content of foods [19]. The total FODMAP consumption of Spanish children aged between 3 and 9 years old was 33.4 g/day, while teenagers between 10 and 17 years old consumed 18% fewer FODMAPS daily. Data about the intake of total FODMAPs in underage population are scarce. As far as we know, Chumpitazi et al. carried out the only clinical trial in a paediatric cohort of young patients (7–17 years old) suffering from IBS, reporting the total FODMAP intake for Typical American Diet in 32.2 g/day (summatory of each FODMAP intake reported) [12]. These data are in agreement with the total FODMAP consumption of Spanish children aged 3–9 years, but not with adolescents’ intake. Apart from the differences in the diet followed by Spanish and American pediatric population, the age of volunteers and their health-status (especially regarding pathologies that affect diet habits) must be taken into consideration. Indeed, age range stratification has been proposed for FODMAP diet research in special populations (infants, pre-school children, primary-school children and high-school children) elsewhere [34].

In addition to child data, the software presented the opportunity to define the daily total mean FODMAP intake by Spanish adults as 21.4 g. These data revealed a significantly lower consumption of FODMAP in this age range than in adolescents or children (reduction of 22% and 36%, respectively). It has been proposed that high-FODMAP foods such as fruits, sweet vegetables, milk, yoghurt- or wheat-based foods are widely consumed among children or adolescents due to their easy acceptation [34]. Our data are in the same line, showing, for instance, an intake of 2.11 g/day of lactose from plain yogurt in Spanish children, while adults consume 1.09 g/day from the same food. That is also the case for apples, whose absolute excess fructose intake was around 1.6 g/day in both age ranges, but whose relative intake (by total energy intake) was higher in Spanish children than in adults. In terms of FODMAP distribution (lactose, fructose excess, oligosaccharides and polyols), the lactose percentage from total FODMAP reduced with age (10% reduction from children to adults), while that of total fructan and excess of fructose was boosted. 

FODMAP intake for adult population in other countries varies from 17.0 to 29.4 g/day. The wide range of FODMAP intake is justified not only by the different dietary habits among countries, but also by other reasons. Some of those researchers analyzed the diet of patients with IBS [23,25], resulting in lower baseline FODMAP intake than the habitual diet of a healthy person. By contrast, even though some other authors showed a similar average consumption of FODMAP, it should be noted that not all of them consistently calculated the total FODMAP content of the diet. As suggested by Varney et al. [17], total a FODMAP analysis must include lactose, fructose in excess of glucose, mannitol, sorbitol, total fructan, stachyose and raffinose. The majority of the research summarized did not measure either the total fructan intake [7,22,30], or the excess of fructose [24].

With the aim of validating the results obtained, we compared our data for adult population with those of Nybacka et al., the most similar research in terms of sample size, total FODMAP classification, or patient’s age [23]. The similarity between Spanish and Swedish FODMAP intake, 21.1 vs. 20.7 g/day, confirmed our approach using national food consumption surveys and placed value on our open software for calculation [19].

In order to better understand the usefulness of the proposed methodology, it is important to note that the LFD strategy is usually based on a list of allowed/not allowed food, considering their total FODMAP content [35,36,37]. While the information provided by that kind of list is necessary [17,38], the “real risk” of each food, also bearing in mind its dietary intake, can be very useful. As in food toxicology, exposure assessments must be determined considering both the chemicals present in foods as well as the food-intake itself. In this line, it must be pointed out that the culture and habits of each country influence the consumption of FODMAPs and its sources [39]. For this reason, we identified top ten foods representing the most important sources of the most consumed FODMAPs (lactose, excess of fructose and total fructan) for Spanish population. This is practical and convenient information for Spanish health-practitioners, for them to use in their daily routine or in their research related to functional abdominal symptoms.

Obviously, the results shown in the present research are limited to the Spanish population. Several authors have proposed data related to different countries or cultures for food composition databases and intake [29,40]. Hence, we would like to highlight the importance of this approach performed to calculate the total FODMAP intake, in order to make its use widespread. The methodology was previously used by others to estimate the presence of inulin and oligofructose in the diets of North-Americans or Europeans [41,42]. GlutenFreeDiet software integrates total fructan, stachyose, raffinose, sorbitol, mannitol, fructose, glucose and lactose content of food. Taking into account that data from National Dietary Surveys are available for most countries, the software allows researchers from other countries to set, without pilot studies, the threshold of FODMAP intake corresponding to their national standard diet. 

Moreover, this software allows fructose excess calculation by meal or by food, when fructose is considered a FODMAP constituent, and not subtracting the total glucose per day to total fructose per day, as calculated in some studies [24,43]. Additionally, the software integrates a wide range of fructan content in food, not usually present in food composition databases. In fact, due to the variability in the FODMAP content of processed foodstuff, and considering that fructans are the most frequent FODMAP found in wheat (one staple food) [44], the total fructan content of eighty-five cereal based processed foods was determined and permanently integrated in the open software for free use [19].

An LFD diet is very limiting and the long-term effects need further evaluation [45]. Among others, nutritional deficiencies concerns surround this kind of diet [46]. Taking this fact into consideration, in the present study we performed an approach to LFD of withdrawing FODMAP risky foods from the diet. The results obtained indicate that the risk of non-compliance for calcium dietary intake was remarkable, followed by fiber, and deficiencies in other vitamins or minerals cannot be discarded. In this line, recent research conducted with IBS patients showed that the number of patients who did not meet thiamin, calcium, cooper and iron recommendations tended to increase after following LFD for 4 weeks (for thiamin, the increase was significant) [47]. It is true that our approximation mirrors the most extreme scenario, in which all the highest lactose, fructose and fructan-risky foods were subtracted from the diet. However, a “top-down” approach for implementing LFD includes an initial phase which restricts all FODMAP subgroups for a 2–8 week period [48]. For this reason, we understand that a complete nutritional software is essential for LFD intervention, in order to control dietary recommendations. 

A clear limitation of this study was the high deviation of the data. Nevertheless, this fact results from the huge sample simple size used (2503 people and 1020 codified foods). By contrast, the applicability (free approach) and the inclusion of total fructan database and intake can be considered a strength of the research.

## 5. Conclusions

In the present study, we demonstrated the relevance of stratifying the total FODMAP intake by age, presenting for the first-time detailed values of their consumption in children and adolescents. In addition to providing a useful and free resource for FODMAP intake quantification, we offer information about FODMAP risk for health practitioners.

## Figures and Tables

**Table 1:** Intake of lactose, excess fructose, total fructan, stachyose, raffinose, sorbitol and mannitol in Spanish population stratified by age.

FODMAP Intake	Children (3–9 Years)		Adolescents (10–17 Years)		Adults (18–74 Years)		Children vs. Adolescents	Children vs. Adults	Adolescents vs. Adults
(g/d)	Mean	SD	Mean	SD	Mean	SD	*p* Value	*p* Value	*p* Value
Lactose	29.1	79.4	22.6	53.9	16.6	41.6	0.06	<0.001	0.01
Excess fructose *	2.43	6.31	2.56	7.04	2.90	7.21	0.70	0.14	0.11
Total fructan	1.13	3.95	1.46	5.09	1.06	4.29	0.15	0.72	0.08
Stachyose	0.02	0.12	0.02	0.15	0.04	0.23	0.99	0.02	0.03
Raffinose	0.06	0.37	0.05	0.37	0.11	0.67	0.59	0.05	0.02
Sorbitol	0.62	2.05	0.57	2.18	0.67	2.52	0.64	0.65	0.38
Mannitol	0.02	0.15	0.03	0.24	0.03	0.17	0.33	0.19	1
Total	33.4	92.3	27.3	69.0	21.4	56.7	0.14	<0.01	0.06

Excess fructose * = fructose−glucose.

**Table 2:** Top ten foods representing the higher intake source of lactose, excess fructose and total fructan in Spanish diet of children population aged between 3 and 9 years old.

Position	Food	Lactose Intake (g/d)		Food	Excess Fructose Intake (g/d) *		Food	Total Fructan Intake (g/d)	
		Mean	SD		Mean	SD		Mean	SD
1st	Whole cow milk	9.33	11.2	Apple	1.52	3.06	White bread	0.14	0.16
2nd	Powdered milk	6.27	31.8	Pear	0.33	1.01	Garlic	0.13	0.17
3rd	Partly skimmed cow milk	5.75	9.54	White bread	0.16	0.18	Onion	0.11	0.16
4th	Plain yogurt	2.11	3.96	Plain sweet biscuit	0.06	0.14	Plain sweet biscuit	0.10	0.22
5th	Flavoured yogurt	1.76	3.17	Orange juice	0.05	0.22	Leek	0.08	0.27
6th	Sponge cake	0.92	4.76	Tomato	0.05	0.10	Sponge cake	0.08	0.37
7th	Skimmed cow milk	0.81	3.65	Melon	0.04	0.21	Sandwich bread	0.08	0.20
8th	Plain skimmed yogurt	0.57	1.45	Honey	0.04	0.23	Chocolate biscuits	0.07	0.24
9th	Egg pudding dessert	0.53	1.58	Orange	0.03	0.12	Chocolate sponge cake	0.02	0.16
10th	Milk chocolate	0.19	0.87	Strawberry	0.03	0.12	Corn flakes	0.02	0.11

* Excess fructose = fructose−glucose.

**Table 3:** Top ten foods representing the higher intake source of lactose, excess fructose and total fructan in Spanish diet of adolescent population aged between 10 and 17 years old.

Position	Food	Lactose Intake (g/d)		Food	Excess Fructose Intake (g/d) *		Food	Total Fructan Intake (g/d)	
		Mean	SD		Mean	SD		Mean	SD
1st	Whole cow milk	7.95	14.44	Apple	1.46	3.20	White bread	0.25	0.24
2nd	Partly skimmed cow milk	7.20	11.19	Pear	0.28	1.02	Onion	0.15	0.24
3rd	Plain yogurt	1.94	4.01	White bread	0.27	0.27	Garlic	0.14	0.24
4th	Skimmed cow milk	1.93	5.92	Tomato	0.08	0.16	Sponge cake	0.10	0.40
5th	Flavoured yogurt	1.30	2.92	Orange juice	0.06	0.27	Leak	0.09	0.38
6th	Egg pudding dessert	0.56	1.68	Orange	0.05	0.14	Plain sweet biscuit	0.08	0.20
7th	Skimmed yogurt	0.40	1.24	Plain sweet biscuit	0.05	0.13	Sandwich bread	0.08	0.25
8th	Powdered milk	0.33	8.19	Honey	0.04	0.25	Chocolate biscuits	0.06	0.25
9th	Flavoured skimmed yogurt	0.11	0.75	Strawberry	0.04	0.15	Pasta	0.06	0.12
10th	Chocolate and hazelnut spread	0.11	0.52	Melon	0.04	0.21	Chocolate sponge cake	0.03	0.20

* Excess fructose = fructose−glucose.

**Table 4:** Top ten foods representing the higher intake source of lactose, excess fructose and total fructan in Spanish diet of adult population.

Position	Food	Lactose Intake (g/d)		Food	Excess Fructose Intake (g/d) *		Food	Total Fructan Intake (g/d)	
		Mean	SD		Mean	SD		Mean	SD
1st	Partly skimmed cow milk	4.87	8.69	Apple	1.78	3.20	White bread	0.17	0.00
2nd	Skimmed cow milk	3.88	7.07	Pear	0.31	1.03	Onion	0.16	0.27
3rd	Whole cow milk	3.57	7.16	White bread	0.19	0.00	Garlic	0.14	0.60
4th	Plain yogurt	1.09	2.80	Tomato	0.12	0.19	Sponge cake	0.08	0.37
5th	Plain skimmed yogurt	0.90	2.11	Honey	0.07	0.31	Leak	0.08	0.38
6th	Flavoured yogurt	0.42	1.87	Orange	0.07	0.15	Sandwich bread	0.06	0.20
7th	Sponge cake	0.32	1.40	Melon	0.05	0.19	Plain sweet biscuit	0.05	0.14
8th	Egg pudding dessert	0.31	1.26	Sweet white wine	0.03	0.36	Peach	0.04	0.19
9th	Flavoured skimmed yogurt	0.28	1.18	Tangerine	0.03	0.10	Watermelon	0.02	0.07
10th	Ice-cream	0.12	0.64	Kiwi fruit	0.03	0.14	Melon	0.02	0.06

* Excess fructose = fructose−glucose.

**Table 5:** Intake change (expressed as percentage of diet contribution) of some micronutrients and fiber after FODMAP risky food subtraction from the diet.

Percentage of Diet Contribution	Children (3–9 Years)	Adolescents (10–17 Years)	Adults (18–74 Years)
Fiber	−55	−47	−55
Vitamin A	−32	−25	−36
Vitamin C	−46	−39	−46
Vitamin D	−14	−9	−5
Riboflavin	−52	−41	−50
Thiamin	−37	−61	−36
Calcium	−72	−59	−68
Magnesium	−44	−38	−41
Zinc	−33	−29	−38
Phosphorus	−45	−39	−43
Iron	−33	−21	−38
